# Reducing the risk of tuberculosis transmission for HCWs in high incidence settings

**DOI:** 10.1186/s13756-021-00975-y

**Published:** 2021-07-19

**Authors:** Ana Paleckyte, Oshani Dissanayake, Stella Mpagama, Marc C. Lipman, Timothy D. McHugh

**Affiliations:** 1 UCL Centre for Clinical Microbiology, Division of Infection & Immunity, UCL, London, UK; 2 Infectious Diseases, Royal Free London NHS Foundation Trust, London, UK; 3 Kibong’oto Infectious Diseases Hospital, Kilimanjaro, Tanzania; 4 UCL Respiratory, Division of Medicine, UCL, London, UK

**Keywords:** Tuberculosis, Transmission, Healthcare workers, LMIC, MDR-TB

## Abstract

Globally, tuberculosis (TB) is a leading cause of death from a single infectious agent. Healthcare workers (HCWs) are at increased risk of hospital-acquired TB infection due to persistent exposure to *Mycobacterium tuberculosis* (*Mtb*) in healthcare settings. The World Health Organization (WHO) has developed an international system of infection prevention and control (IPC) interventions to interrupt the cycle of nosocomial TB transmission. The guidelines on TB IPC have proposed a comprehensive hierarchy of three core practices, comprising: administrative controls, environmental controls, and personal respiratory protection. However, the implementation of most recommendations goes beyond minimal physical and organisational requirements and thus cannot be appropriately introduced in resource-constrained settings and areas of high TB incidence. In many low- and middle-income countries (LMICs) the lack of knowledge, expertise and practice on TB IPC is a major barrier to the implementation of essential interventions. HCWs often underestimate the risk of airborne *Mtb* dissemination during tidal breathing. The lack of required expertise and funding to design, install and maintain the environmental control systems can lead to inadequate dilution of infectious particles in the air, and in turn, increase the risk of TB dissemination. Insufficient supply of particulate respirators and lack of direction on the re-use of respiratory protection is associated with unsafe working practices and increased risk of TB transmission between patients and HCWs. Delayed diagnosis and initiation of treatment are commonly influenced by the effectiveness of healthcare systems to identify TB patients, and the availability of rapid molecular diagnostic tools. Failure to recognise resistance to first-line drugs contributes to the emergence of drug-resistant *Mtb* strains, including multidrug-resistant and extensively drug-resistant *Mtb*. Future guideline development must consider the social, economic, cultural and climatic conditions to ensure that recommended control measures can be implemented in not only high-income countries, but more importantly low-income, high TB burden settings. Urgent action and more ambitious investments are needed at both regional and national levels to get back on track to reach the global TB targets, especially in the context of the COVID-19 pandemic.

## Background

Tuberculosis (TB) remains one of the leading causes of preventable morbidity and mortality worldwide with approximately 10 million new cases (estimated range 8.9–11 million) and 1.4 million deaths (estimated range 1.2–1.5 million) in 2019 [1].

From 2020, the Coronavirus disease 2019 (COVID-19) pandemic has contributed to severe disruption to essential TB care, services, and infectious disease epidemiology. For example, the provisional TB case notification data for 2020 was reported from only 84 countries, whereas in 2019 this was 198 countries accounting for 99% of the estimated global TB disease. Estimates suggest that there will be marked reductions in TB detection that translate into 21% of individuals with suspected TB infection not having received medical care in 2020 [2]. The World Health Organization (WHO) estimates that COVID-19 will result in half a million excess TB deaths. The current pandemic threatens to reverse recent achievements in TB control towards the 2025 milestone of the End TB Strategy. Considerable and prompt action is needed, therefore, if we are to reach the intended global targets [1–3].

The WHO recommends systematic monitoring of high-risk population groups, including household contacts of TB-affected individuals, people living with human immunodeficiency virus (HIV) infection and healthcare workers (HCWs). HCWs play a central role in global TB elimination, yet their contribution is undermined by the risk of hospital-acquired TB [4]. Nosocomial *Mycobacterium tuberculosis* (*Mtb*) infection is increasingly recognised in high TB burden and low- and middle-income countries (LMICs), which account for 87% of global cases of TB disease [1]. The number of TB cases per 100 000 HCWs in some LMICs is more than double the incidence rate among the general population, implying that healthcare facilities are an important source of TB transmission in these countries [1, 4].

Nosocomial transmission of drug-resistant TB (DR-TB), defined as *Mtb* resistant to at least one first-line drug, is a major public health concern. Multidrug-resistant TB (MDR-TB) is characterised by in vitro resistance to both first-line drugs, rifampicin and isoniazid. Extensively drug-resistant TB (XDR-TB) was initially defined as MDR-TB with additional resistance to both fluoroquinolones and second-line injectables [1]. However, the definition was revised by WHO and implemented in early 2021 due to recent changes in DR-TB treatment regimens and diagnostics. Injectable agents are no longer prioritised for the treatment of rifampicin-resistant (RR) and MDR-TB patients, and thus have been replaced by more effective oral antibiotics with less associated adverse effects. The new or repurposed antibiotics, including fluoroquinolones, bedaquiline and linezolid, belong to Group A drugs that are currently recommended for MDR-TB regimens, whenever such regimens can be used. Therefore, in the updated definition, XDR-TB refers to MDR/RR-TB strains that are also resistant to any fluoroquinolone and at least one additional Group A drug [5].

The transmission of drug-resistant *Mtb* strains in healthcare settings is well documented [1, 4, 6]. In 2005, the largest outbreak of XDR-TB identified in Tugela Ferry, South Africa alerted the world to the prospect of transmission of potentially untreatable TB [6]. The predominant strain (LAM4) was resistant to at least four classes of antibiotics, leading to fatal TB disease, with 98% mortality among HIV-coinfected patients. Following investigation, Cohen et al. [6] reported that the emergence of resistant *Mtb* strains commonly occurs as a result of undetected resistance mutation to first-line drugs, followed by prescription of inappropriate treatment regimens. The initial pattern of drug resistance usually cannot be optimally detected by available molecular diagnostic tools, and as a result, subsequent accumulation of compensatory mutations drove the development of MDR and XDR strains, which continue to circulate today.

TB infection prevention and control (IPC) is one of the key components of the End TB Strategy applied at both the facility and national level to prevent the spread of TB disease, including its drug resistant forms [1, 3]. Since 1999, an international system of IPC interventions has been developed and updated by the WHO to reduce the risk of transmission and exposure to *Mtb* in healthcare settings. Guidelines and policies on IPC have been proposed as an integrated package of three core practices aimed at achieving an integrated, patient-centred care and supportive systems [3]. Although IPC strategies and interventions can be easily implemented in high-income countries, there is limited evidence available regarding the feasibility of such IPC measures in resource-constrained and high TB burden areas [4]. This review seeks to document challenges in current international TB IPC and outlines the potential strategies to prevent the spread of infectious pathogen across different levels of healthcare, particularly in LMICs and high TB burden countries (HBCs).

## TB IPC measures in healthcare settings

The WHO multimodal IPC strategy consists of a combination of interventions designed to minimise and prevent the risk of *Mtb* transmission in healthcare settings. It had been suggested that IPC measures should not be prioritised individually or adopted separately, but must be considered as an integrated package of controls [3]. A three-tiered approach of measures comprising of administrative controls, environmental controls, and personal respiratory protection (Table 1) is recommended for implementation across healthcare settings.

**Table 1:** Summary of IPC interventions based on a three-level hierarchy of controls

Administrative controls	Environmental controls	Personal respiratory protection
Triage and isolation of people *(with presumed or confirmed TB infection)*	Ventilation systems Natural Mechanical Mixed-mode Recirculated air through HEPA filters	Particulate respirators (N95 or FFP2)
Prompt initiation of effective treatment		
Respiratory hygiene *(including cough etiquette)*	GUV systems Germicidal lamps Upper-room GUV	Respirator fit testing
Management of HCWs *(including education and training)*		

The recommendations listed above were summarised from the WHO 2019 guidelines on TB IPC [3]. HCWs, healthcare workers; HEPA, high-efficiency particulate air (filters); GUV, germicidal ultraviolet

### Administrative controls

Administrative controls are the most important level of IPC hierarchy, and includes triage and isolation of patients with suspected or confirmed TB infection, prompt diagnosis and initiation of treatment, promotion of respiratory hygiene and management of healthcare personnel (i.e. education and training of HCWs) [3, 7]. These measures should be considered as the primary IPC standard that defines the quality of TB service delivery in healthcare settings. Although the recommended interventions can be effectively adopted and maintained in high-income countries, they cannot always be appropriately or easily translated into practice in many high-burden LMICs as there can be limitations to required resources to effectively implement procedures in such settings [4, 8].

#### Triage and isolation

From the onset, many facilities may lack the ability to effectively triage and isolate patients. A study conducted by Naidoo et al. [9] revealed that of the 51 primary healthcare clinics in KwaZulu-Natal, South Africa, only 26% practiced triaging of coughing patients, and 2% were able to accommodate patients thought likely to have TB in a separate room. Many publications have highlighted issues of availability of designated isolation facilities in hospitals that provide specialised TB services. Inappropriate allocation of space for triage and isolation is challenged by overcrowding and a high turnover of patients in these countries [10, 11]. Bed occupancy commonly exceeds the standard capacity of the healthcare facility. As an outcome, infectious TB patients can stay amongst other hospitalised individuals for a duration of time prior to isolation rooms becoming available, if at all.

Our own work has found that the lack of knowledge to identify MDR-TB infectious cases contributes to delayed triage and isolation of TB patients [12]. Another major challenge with triaging is the separation of new TB patients from those who have already started the treatment. New patients are most likely to be bacteriologically positive and infectious. By contrast, treated patients may become bacteriologically negative and enter the convalescence stage during the treatment. It has been reported that the median time to culture conversion among Tanzanian patients on treatment is 6 weeks[ 13]. To address this issue, the Kibong’oto Infectious Diseases Hospital, Tanzania has built specific MDR-TB wards. Following the reconstruction, new patients are admitted to MDR-TB infectious ward, where each isolation room can accommodate only two patients. A convalescence ward was designed to accommodate recovering, bacteriologically negative MDR-TB patients, from 8 to 12 individuals per room. MDR-TB patients treated in these wards are referred from other healthcare settings located in different parts of the country, where the implementation of such practice remains a significant challenge.

#### Prompt initiation of effective treatment

Timely initiation of effective treatment for TB relies mainly on two factors: individual patient behaviour to seek advice after becoming symptomatic and the effectiveness of healthcare systems to correctly identify TB patients and refer them to specialised TB centres for diagnosis and treatment [1, 3]. The “delay” from TB symptom onset to final diagnosis varies from country to country. A study conducted in urban Uganda found that the median total delay to TB diagnosis was 70 days [14]. Diagnostic delay in urban China was reported as being approximately 50 days [15]. Just as importantly, patients residing in rural areas of Ethiopia demonstrate a three-fold increase in the median length of a delay compared to those from urban areas [16]. Sekandi et al. [14] reported that the initial decision to seek care from healthcare providers is negatively influenced by low education, poverty, old age, alcohol use and lack of access to healthcare settings. Another important cultural factor of delayed diagnosis is the stigma, which drives people to conceal their disease and avoid being screened for TB by healthcare professionals [15]. Moreover, the delays also occur through interaction with non-TB heath providers, who are not familiar with the standard screening algorithms for symptomatic patients and thus, cannot refer a patient to specialised TB services [14, 15].

Clinical factors, such as negative acid-fast bacilli (AFB) smears and absence of typical cavitary lesions on chest radiographs, also significantly delay the confirmation of TB infection and commencement of treatment [15–18]. Moreover, a clear association between treatment delay, clinical severity and increased transmission of the disease has been previously described [19, 20]. Failure to begin timely treatment can lead to destructive damage to lung parenchyma and the development of pulmonary cavities, containing very high mycobacterial burden. There is a sufficient level of evidence that cavitation accompanied by sputum smear positivity correlate with increased infectiousness and disease transmissibility [20]. Severe pulmonary cavitation can also lead to weak therapeutic response and emergence of drug resistance. In a cohort study of patients with pulmonary MDR- and XDR-TB, there was a high proportion of individuals who had additional drug resistance in *Mtb* isolates from cavitary lesions compared with sputum [21]. The data suggested that the development of drug resistance occurs due to high bacterial loads and potentially low drug concentrations in tuberculous cavities. These findings demonstrate that delayed diagnosis and treatment can lead to the development of drug resistant strains and promote the transmission of highly infectious pathogens at both healthcare and community levels [22].

Cohen argued that the emergence of drug resistance commonly occurs not due to delayed initiation of effective TB treatment but instead to limited availability of rapid molecular diagnostics that allow the initial emergence of drug resistance to be undetected [6]. Current diagnostic algorithms focus on early detection of rifampicin resistance as a marker of MDR-TB and do not include a routine detection of resistance to other antitubercular compounds [1, 3]. Although some rapid molecular diagnostic tools, such as GeneXpert, became available in many high TB burden and LMICs, the testing coverage for isoniazid remains low [1, 6]. This means that an important group of TB patients susceptible to rifampicin but resistant to isoniazid are not identified and consequently treated with an inappropriate drug regimen. Failure to recognise isoniazid resistance is clearly exemplified by the historical XDR-TB outbreak identified in Tugela Ferry [6]. The genomic analysis of clinical *Mtb* isolates revealed that resistance to isoniazid was overwhelmingly the first resistance mutation to be acquired along the pathway to multiple drug resistances. Authors provided strong evidence that isoniazid resistance is a key initiation event towards emergence of MDR and XDR strains, and therefore, this drug resistance pattern should be checked prior to initiation of any TB treatment [1, 6].

#### Respiratory hygiene

The WHO strongly recommends respiratory hygiene techniques for individuals suspected or confirmed as having TB. Respiratory hygiene (including cough etiquette) is defined as the practice of covering of the mouth and nose during coughing and sneezing. This includes the recommendation of surgical or cloth masks for such patients in order to reduce dispersal of respiratory secretions containing infectious particles [1].

Cough is commonly assumed to be a primary mechanism of TB transmission. However, the risk of producing and transmitting infectious *Mtb* aerosols during tidal breathing is underestimated [23, 24]. Since *Mtb* bacillus is 0.2–0.5 µm wide and 2–4 µm long, it can be effectively transmitted by bioaerosols in the 1–5 µm range. A patient with persistent cough predominately produces large droplets (> 5 µm). Whereas, smaller bioaerosols measuring < 5 µm are generated during normal tidal breathing. Indeed, smaller particles, once inhaled, demonstrate a higher probability of reaching and depositing in the lower respiratory tract than those of > 5 µm [23]. A study conducted by Wurie et al. [24] showed that intrathoracic TB was associated with higher particle production, with nearly fourfold increase in odds of production of small bioaerosols compared with healthy individuals. However, further investigation is needed to determine whether high bioaerosol production during normal breathing contributes towards increased infectiousness.

Current findings have implications for the risk assessment of suspected and confirmed TB patients, and highlight the importance of personal safety measures undertaken by HCWs and other susceptible contacts. The promotion of respiratory hygiene is a relatively simple measure, which is generally not limited by funding, unlike a number of the other measures described. However, it relies upon education and training amongst HCWs, if these measures are to be implemented successfully in practice.

#### Management of healthcare workers

The lack of education and training activities on infection control policies and other essential work practices remains a major barrier to effective implementation of TB control measures in LMICs. According to a study conducted in a large academic hospital in Cape Town, South Africa, a significant and high proportion of HCWs had a poor level of knowledge and practice regarding TB IPC [8]. Of the 20 nurses who participated in interviews, none were familiar with the SA National TB guidelines and none had received any TB IPC-related training. The majority of participants had misconceptions about the time period during which TB patients remain infectious following initiation of anti-tubercular therapy. Nurses also were unable to identify different types of respiratory protection and wrongly believed that surgical masks can protect HCWs from inhaling infectious aerosols [8]. Findings by Gizaw et al. [25] revealed that HCWs in selected public health facilities in Addis Abba, Ethiopia had a relatively good overall knowledge of TB IPC compared to survey results of hospital staff in Cape Town [8]. Around 91% of the respondents understood the importance of triaging patients with suspected TB disease and almost 90% were aware of the importance of respiratory hygiene techniques. Although nearly two-thirds of study participants demonstrated good theoretical knowledge about TB infection control, half of them had unsatisfactory practice in TB IPC. This was primarily associated with a low proportion, around 40%, of experienced and trained HCWs in these facilities [25].

Garnett et al. [26] reported that laboratory workers were also at high risk of acquiring TB infection. Despite the least contact with patients, laboratory staff had a persistent exposure to TB specimens. National Health Laboratory Service (NHLS) in South Africa collected data between 2012 and 2019 to evaluate the risk of TB within different occupational groups [26]. Laboratory workers had a higher incidence rate (160 per 100,000 person-years) compared to medical staff (133 per 100,000 person-years). The lack of training, knowledge and awareness of TB transmission was identified as the main factors for high disease incidence in this group. This was also linked to inappropriate sample handling and good laboratory practice not being followed [26]. These findings suggest that clinical and non-clinical HCWs may have different levels of education and TB IPC training, which consequently contribute to their ability to recognise and respond to potential *Mtb* exposure.

### Environmental controls

Environmental controls are intended to reduce the concentration of infectious *Mtb* droplet nuclei in the air through the dilution, filtration and disinfection principles. According to WHO recommendations, this can be achieved through appropriate commissioning of specialised ventilation and Germicidal Ultraviolet (GUV) systems [27]. However, correct installation, maintenance and ensuring overall sustainability of such intervention requires allocation of sufficient resources, which can be limited in LMICs.

#### Ventilation systems

Effective monitoring of *Mtb* droplet nuclei concentration levels in the air is particularly important to prevent airborne transmission in high TB burden settings. Ventilation systems, including natural, mechanical, mixed-mode ventilation, and recirculated air through high-efficiency particulate air (HEPA) filters maximise airflow rates to dilute and/or filter potentially infectious particles in the air [3]. These systems are also implemented to generate negative pressure gradients to prevent the spread of airborne *Mtb* outside of the settings, such as airborne infection isolation rooms, where the risk of transmission is high. However, in most resource-constrained healthcare settings, natural ventilation is considered to be the only affordable environmental TB IPC measure. Many hospitals in South Africa have a very limited number of specialised isolation rooms and no artificial ventilation [11].

In tropical or temperate climates, natural ventilation is a low-cost alternative, which has the advantage of wide availability and relatively high efficacy. Occasionally, TB patients are cared for in hospital veranda or corridors with open windows to allow the airflow from the outside to dilute *Mtb* bacilli in the air [11, 28]. A recent study demonstrated that natural ventilation can be improved through simple architectural modifications to existing healthcare facilities, reducing the risk of TB transmission for HCWs and patients by 72% [27]. However, this environmental measure is usually not feasible in colder climates and particularly during winter. Healthcare settings in countries with limited ability to consistently use natural ventilation should consider artificial alternatives to ensure effective dilution of infectious *Mtb* droplet nuclei in the air [3].

The lack of required expertise and funding may impede the implementation of specialised ventilation systems. It should be noted that the use of poorly designed and maintained ventilation can lead to inadequate airflow and disruption of differential air pressure, which in turn increases the risk of TB transmission within healthcare facilities and congregate settings, such as correctional facilities [3, 29]. The importance of negative pressure in rooms used for containment isolation of TB patients has been clearly illustrated by MDR-TB outbreak occurred at St. Thomas’s Hospital, London [30]. A patient with MDR-TB was admitted to the isolation room, which unbeknown to hospital staff, was at positive pressure relative to the main ward. HIV patients were nursed in side-rooms of this ward, and as an outcome, seven of the 64 HIV-positive inpatient contacts developed MDR-TB. Following the investigation, inadequate airflow in isolation rooms was defined as a major factor contributing to this outbreak.

It is of paramount importance that use of ventilation systems are correct in that they continue to provide sufficient dilution and removal of infectious particles. Without proper installation and maintenance, this control measure can be costly and ineffective. It is essential for IPC teams to know the airflow characteristics of all isolation rooms in their healthcare institutions. The management and good communication between IPC team, clinicians and nursing staff is also important to achieve appropriate functioning of IPC interventions.

#### Germicidal ultraviolet systems

GUV systems, including lamps and upper-room installations, are recommended for use to disinfect the air in healthcare facilities [3, 27]. If the local healthcare authorities are able to allocate the resources for installation of ventilation and/or GUV systems, hospitals in LMICs frequently face subsequent challenges. Many studies have reported issues of being unable to install, perform quality control testing, and maintain ventilation and disinfection units. HCWs and other hospital staff are not trained and thus, cannot undertake the necessary maintenance and fixing of these systems. Moreover, many hospitals are also unable to cover the costs of regular assessment of technical performance, and as a result, the installed systems remain non-functional for several months or even years [27–29].

### Personal respiratory protection

Respiratory protection controls consist of the use of personal protective equipment (PPE) in instances that pose a high risk of exposure to *Mtb*. Particulate respirators (N95 or FFP2) with a filter efficiency level of at least 94% against > 0.3 µm particles should be fit-tested, by appropriately trained individuals, prior to use to prevent incorrect use and a false sense of protection. Correctly-fitted respirators should be worn when in contact with suspected or known infectious TB patients, and especially during aerosol-generating procedures, such as sputum induction or bronchoscopy [3, 31].

#### Particulate respirators

The use of particulate respirators by HCWs is considered to be the most important personal safety measure that should be undertaken in instances when the risk of TB transmission is high [3]. However, the study conducted by Parmar et al. [32] demonstrated that routine wearing of PPE in resource-constrained Indian healthcare settings with specific high-TB risk departments was observed in only 10% of hospitals. The literature suggests a number of limiting factors to the correct usage and donning of respirator, including a lack of supply, funding, education and training. In many settings, disposable N95 respirators are available on TB wards, but are reserved only for use in drug-resistant TB cases. Similarly, studies have highlighted that the use of simple surgical masks are usually reserved only for patients with drug-resistant TB and are rarely given to those with drug-susceptible or suspected TB disease [8, 12]. Many healthcare facilities lack financial support to provide single-use PPE to all HCWs, as well as all suspected and infected patients.

There is currently a limited body of evidence or guidelines pertaining to the re-use and storage of disposable respiratory masks, although this is common practice in many healthcare settings. Zinatsa et al. [33] conducted a study in 43 primary healthcare facilities in Mangaung, South Africa and reported that many staff members did not know the recommended duration of respirator usage. Some quoted usage of disposal respirators up to several months or until they become loose and dirty. This practice reflects a lack of direction on the re-use of respiratory protection and training at local and individual levels.

#### Respirator fit testing

Despite the availability of PPE in some healthcare settings, TB IPC measures commonly do not fully comply with international standards due to a lack of fit testing prior to use [3, 33]. This may be as a result of a number of factors, including a lack of training equipment and expertise to provide constant training to staff and HCWs. Due to potential shortage of respiratory protection supply in many settings, the donning of respirators can often be perceived as optional for HCWs during aerosol-generating procedures, such as sputum induction and bronchoscopy. However, there is an underestimated risk of unexpected *Mtb* exposure during such procedures, even when performed on AFB smear negative individuals. It has been reported that approximately 5% of patients, not initially suspected as presenting with TB are subsequently diagnosed with it following bronchoscopy [34]. This demonstrates that lack of respiratory precautions during aerosol-generating procedures, and when caring for TB patients, increases HCWs’ risk of acquiring and transmitting TB.

## TB infection control data

### Understanding Mtb transmission

A sequencing-based approach is commonly applied for characterisation of *Mtb* strains and identification of drug-susceptibility patterns [35, 36]. We demonstrated that sequence analysis of resistance genes can also support tracing of infectious cases [36]. The similarity of the isolates obtained from the previously described St. Thomas’s Hospital outbreak was investigated by IS6110 and polymorphic repetitive sequence typing. Sequencing results revealed that all isolates were genetically indistinguishable, therefore providing the clinical evidence of a single *Mtb* strain transmission amongst hospital patients [36]. Moreover, genotyping of *Mtb* isolates can be performed in cases of recurrent TB infection. The report by Murphy et al. [37] highlighted its value in determining whether recurrent TB in a healthcare worker occurred from reactivation of previous disease or through new infection. Sequencing showed that the new isolate differed by nine loci from the historical isolate, thus excluding TB relapse. However, it matched an isolate in the National Mycobacterial Reference Laboratory database sequenced from a person living with HIV who had been diagnosed with pulmonary TB whilst an inpatient in the hospital where the HCW worked. The patient had spent less than 20 min in the same room as the HCW during his hospital admission. The HCW had a higher risk for TB disease due to ongoing treatment with an anti-tumour necrosis factor (anti-TNF) agent being taken for another condition.

The role of whole genome sequencing (WGS) in identifying the transmission networks of DR-TB has been recently described [35, 38, 39]. The WGS-based approach employed in the study conducted by Williams et al. [39] provided insights into the dynamics of MDR-TB transmission between low and high TB burden areas. An HIV-negative patient 1, with no history of travel abroad, has been admitted to the UK hospital with MDR-TB. To investigate the origin of the infection, WGS-relatedness analysis was performed using both mycobacterial interspersed repetitive unit-variable number tandem repeat (MIRU-VNTR) and single nucleotide polymorphism (SNP)-based calculation of distances. The distance difference of ≤ 5 SNPs between the isolates obtained from patients with suspected epidemiological links was used to indicate recent TB transmission [35, 39]. A matching genomic profile was identified from an HIV-positive HCW who received TB treatment in the same hospital (patient 2). They had previously worked at Tugela Ferry Hospital, South Africa, which was associated with the largest outbreak of XDR-TB [6]. The results were compared with a national UK database and matched two other isolates obtained from African-born HCWs (patients 3 and 4) admitted to another UK hospital. Sequencing libraries from these 4 UK *Mtb* isolates were then compared with 36 South African strains, including one from the Tugela Ferry outbreak (KZN605). According to pairwise comparison of WGS data, patients 1 and 2 were infected with a strain closely related to KZN605. Moreover, the sequences from these two patients differed by only 4 SNPs, suggesting a high probability of nosocomial TB transmission. Just as importantly, WGS-based analysis revealed that *Mtb* isolates from patients 3 and 4 were associated with another strain circulating in South Africa with a difference of 69–72 SNPs compared to the isolates obtained from the first patient pair.

A WGS-based approach can not only identify and monitor the emergence of TB outbreaks at a healthcare facility or community level, but also has the potential to efficiently elucidate transmission events at a national and international level. The establishment of centralised WGS-based surveillance systems for TB would allow the development of a registries and database to better understand the dynamics of *Mtb* transmission across different countries.

### Evidence on TB IPC interventions

The effectiveness of IPC interventions in reducing transmission of *Mtb* among HCWs and persons attending healthcare settings has been estimated in a number of studies [3, 40]. A systematic review conducted by Fielding et al. [41] concluded that HCWs had twice the odds of TB infection when compared to the general population, with similar rates when stratified for high and low TB burden countries. However, it is clear that TB disease incidence data from meta-analyses must be interpreted with some caution as there is much heterogeneity across the studies used to build the body of evidence currently available [41]. The evidence for effectiveness of IPC recommendations, alone and in combination, remains generally indirect and of low quality. Many studies are observational in nature, some with a high risk of bias and confounding, including design specific issues and heterogeneity of the results [40–42]. Overall assessment and guidance is further constrained by the limited availability of data from high TB burden healthcare settings. Of the 25 selected primary research reports, 76% were conducted in high-income, low TB burden countries, predominantly in the United States [42]. Although there is limited evidence for effectiveness of IPC measures, overall, the implementation of multiple controls demonstrated an absolute risk reduction for TB incidence among HCWs and individuals attending the healthcare facilities [42, 43]. The extent of the risks of acquiring TB has also been carefully assessed, and a strong priority was assigned to the systematic introduction of core practices alongside other IPC recommendations [3, 41]. The WHO guidelines stress that their recommendations are based on the effect of composite measures, and therefore accurate assessment of the effect of single aspects of recommended practices was not possible.

### Future directions

Current control measures are strongly recommended by the national health authorities and must be adopted in all healthcare settings that pose a high risk of nosocomial TB transmission [4, 42]. Implementation of good infection control policy and practice requires buy-in at not only a healthcare facility level, but also a subnational and national level. TB control programs must strengthen TB education and improve awareness of the signs and symptoms of TB within communities. HCWs, including general healthcare professionals as well as TB-service providers, should complete continuing medical education and TB IPC-specific training to be able to suspect, identify and promptly diagnose TB patients. Moreover, the diagnostic algorithms currently employed in high TB burden and LMICs should include routine testing for both rifampicin and isoniazid resistance to ensure early detection of DR-TB.

The IPC measures are even more pertinent in the context of the COVID-19 pandemic. As the world now learns to live with this disease, we are aware of its impact on our management of other infections, such as the TB pandemic. Since early 2020, COVID-19 has caused severe disruption to vital TB services, disproportionately affecting people in LMICs, who are already at greater risk for developing TB [2]. Negative impacts on access to TB care and treatment have been reported by 184 countries [1, 44]. Many described the reallocation of TB resources to the COVID-19 response. This included the reassignment of TB clinicians, nurses and other staff to COVID-19-related duties (85 countries, including 20 high TB burden countries), the use of laboratory equipment (e.g. GeneXpert) for COVID-19 testing instead of diagnostic TB testing (43 countries, including 13 with high TB incidence), and reallocation of TB funding (reported by 52 countries). Moreover, up to 70% of countries involved in WHO’s global TB data collection for 2020, reported reductions in the number of outpatient visits for individuals with TB disease [1]. The procurement and transportation of laboratory consumables, medicines, PPE and other essential resources have been also disrupted, negatively affecting TB services and maintenance of TB IPC measures [1, 44]. Inevitably worse IPC for TB will lead to greater transmission of other respiratory infections. The use of measures such as lockdown (to keep households together) will lead to more infection transmission of TB in these settings unless IPC is strictly applied. This feels somewhat of a forlorn hope given the economic, social and psychological pressures that many families are currently experiencing.

Modelling studies have estimated the number of excess deaths from TB, and concluded that over a five-year period in high burden settings death could increase by 20% [45]. There is growing anxiety about the impact on new infection rates, the potential propagation of DR-TB and the synergistic effect of COVID-19 and TB co-infection. A rapid modelling assessment by Stop TB partnership predicted that the number of excess deaths between 2020–2025 that would arise for every month taken to return to normal TB service delivery, would be in India, an additional 40,685 deaths, 1,157 in Kenya and 137 in Ukraine [46]. As COVID-19 continues to affect many parts of the world, though disproportionately in LMICs, the emphasis on strategies focusing on prevention of transmission is of increasing importance.

The WHO has developed the new policies and guidelines to maintain continuity of healthcare services for TB patients affected during the COVID-19 pandemic [1, 47]. These include ensuring accurate diagnosis of both TB and COVID-19. The Stop TB partnership proposes simultaneous, integrated testing, which can be performed using nucleic acid amplification tests (NAATs). Studies suggest that the presence or history of *Mtb* infection is associated with severe COVID-19 symptom development, leading to life-threatening complications and poor outcomes, especially in cases of TB/COVID-19 co-infection [48, 49]. The diagnostic testing using GeneXpert, Abbott Realtime and Roche Cobas 6800/8800 platforms is particularly important for countries where the risk of co-infection is elevated. The procurement of such systems for high TB burden countries is currently supported by WHO and the Global Fund [48].

Healthcare services are also reducing the number of outpatient visits for *Mtb* infected individuals [1, 2]. Promoting access to community-based prevention and care to reduce transmission is a priority. This includes treatment at home, allowing TB patients to collect a monthly supply of drugs from healthcare providers, or nominate another household member to receive medicines on their behalf. It is also supported through the use of WHO-recommended all-oral TB drugs for patients with MDR and XDR-TB [1, 2, 47]. Moreover, the adherence to digital technologies must be promoted and enhanced to support TB patients through improved communication, remote care and advice, and information management. Modern digital interventions, such as electronic directly observed therapy (eDOT), are commonly used for initial diagnostic assessments, TB patient supervision and treatment adherence. The eDOT, which can be accessed through mobile apps, video calls and text messages, is effectively practiced in some high TB burden countries, including China and India [50]. In line with WHO recommendations, electronic healthcare services could help bridge the communication gap and enhance TB care and control, for HCWs and patients, with little increased risk of infection transmission,. However, such interventions need to be matched with adequate human resources, support from funding bodies and political commitment [1, 47, 50].

Limiting the transmission of both TB and COVID-19 in congregate settings and healthcare facilities requires effective IPC measures. TB program staff, including doctors, nurses and the IPC team, should familiarise themselves with the most recent WHO recommendations for prevention, detection, care and treatment of both TB and COVID-19 [51]. Governments and healthcare facility managers need to be made aware that they are responsible for providing information, instruction and training on IPC to ensure that HCWs are operating under effective safety standards. Personnel must be trained to be able to assess, diagnose, triage and treat patients, and share IPC information with patients and the public [47]. It is also important to provide all necessary protective measures, including good-quality PPE (e.g. gowns, gloves, googles, respirators and hand sanitisers), which are used in line with the latest WHO guidelines. The implementation of such interventions should be assessed and evaluated by through IPC assessments. Internal and external audits must be periodically conducted in accordance with current best practice guidelines and commissioning toolkits, such as NICE [47, 51]. National TB program partners should systematically measure and report their TB quality care results, with the aim of driving up care, especially in low-income, high TB burden countries, where healthcare services quality may fall short of international standards [1].

Appropriate planning and budgeting for both TB and COVID-19 are essential to ensure that procurement and delivery of TB drugs and diagnostics supplies are not interrupted [47]. The collaboration of key partners, including WHO, the Global Fund, the Stop TB partnership and USAID, is essential in supporting the most vulnerable countries by securing adequate and sustainable supplies for TB healthcare services [1, 2, 47]. The mobilisation of additional funding parties will be critical in the near future as new investments are urgently required to catch up with most TB care activities during any COVID-19 recovery stage [47]. Through this it is hoped that countries will be able to reinstate and progress their national TB plans, and so achieve the global targets set out within the End TB Strategy.

## Conclusion

Future guideline development relies on the body of evidence available. Unfortunately, what we have for the prevention and control of TB is some way from that needed to effectively manage the global burden of disease and TB transmission in healthcare settings. There remains a need for more robust studies that can support evidence-based guideline development. These must also consider the social, psychological, economic, cultural and climatic conditions encountered in not only high-income settings, but more importantly low-income, high burden TB endemic countries if IPC strategies are to be effective in practice. It is crucial to strengthen and maintain TB services in order to achieve universal health coverage with resilient systems, that can ensure effective synergistic responses to TB and COVID-19, as well as other pandemics that may arise in future.

## Data Availability

Are available on request to TMcH.

## Abbreviations

AFB: Acid-fast bacilli
Anti-TNF: Anti-tumour necrosis factor
COVID-19: Coronavirus disease 2019
DR-TB: Drug resistant TB
eDOT: Electronic directly observed therapy
GUV: Germicidal ultraviolet
HCW: Healthcare worker
HEPA: High-efficiency particulate air
HIV: Human immunodeficiency virus
IPC: Infection prevention and control
LMIC: Low- and middle-income country
MDR-TB: Multidrug-resistant TB
MDR/RR-TB: Multidrug-resistant or rifampicin-resistant TB
*Mtb*: *Mycobacterium* *tuberculosis*
NAAT: Nucleic acid amplification test
NICE: National Institute for Health and Care Excellence
PPE: Personal protective equipment
RR-TB: Rifampicin-resistant TB
SNP: Single nucleotide polymorphism
TB: Tuberculosis
USAID: United States Agency for International Development
WGS: Whole genome sequencing
WHO: World Health Organization
XDR-TB: Extensively drug-resistant TB
